# Ultrasound Versus Magnetic Resonance Imaging in the Evaluation of Shoulder Pain in End Stage Renal Disease Patients on Chronic Hemodialysis

**DOI:** 10.1155/2022/1315446

**Published:** 2022-10-28

**Authors:** Samar Tharwat, Eman Nagy, Mohamed Mohsen, Mohammed Kamal Nassar

**Affiliations:** ^1^Rheumatology and Immunology Unit, Internal Medicine Department, Faculty of Medicine, Mansoura University, Mansoura, Egypt; ^2^Mansoura Nephrology & Dialysis Unit (MNDU), Department of Internal Medicine, Faculty of Medicine, Mansoura University, Mansoura, Egypt; ^3^Radiodiagnosis Department, Faculty of Medicine, Mansoura University, Mansoura, Egypt

## Abstract

**Background:**

Musculoskeletal pain is common in hemodialysis (HD) patients and may be related to articular or periarticular amyloid deposition. The shoulder is one of the most common afflicted joints, but not all causes of shoulder pain are detectable on radiography, and magnetic resonance imaging (MRI) is not always available. The aim of this study was to evaluate the validity of musculoskeletal ultrasound (MSUS) to properly detect shoulder disorders in HD patients by identifying US abnormalities in the shoulder and comparing them to those identified on MRI, with MRI serving as the gold standard test.

**Methods:**

This cross-sectional observational study was conducted on 28 HD patients (16 males and 12 females, mean age 46.89) with either unilateral or bilateral shoulder pain. Demographic data and clinical characteristics were recruited. All patients were subjected to clinical assessment, MSUS and MRI of both shoulders.

**Results:**

US abnormalities were prevalent in almost all patients. Supraspinatus tendinopathy was the most common abnormality in symptomatic shoulders (92.1%), followed by subacromial-subdeltoid (SASD) bursitis (65.8%), humoral erosions (57.9%), and acromioclavicular joint (ACJ) osteoarthritis (52.6%). MSUS shows high sensitivity and specificity when compared to MRI in all the studied shoulder pathologies except glenohumeral joint (GHJ) effusion (sensitivity, 33.3%) and infraspinatus tendinopathy (sensitivity, 58.3%). The percentage of agreement between MSUS and MRI in detecting biceps tenosynovitis was 82.14% (kappa, 0.64), subscapularis tendinopathy 83.93% (kappa, 0.654), supraspinatus tendinopathy 91.07% (kappa, 0.617), infraspinatus tendinopathy 82.14% (kappa, 0.470), SASD bursitis 80.36% (kappa, 0.569), humeral head erosions 82.14% (kappa, 0.635), GHJ effusion 82.14% (kappa, 0.352), and ACJ osteoarthritis 76.79% (kappa, 0.539).

**Conclusions:**

Shoulder problems are common in HD patients, even in people who do not have obvious shoulder complaints. MSUS is a valuable imaging technique that assists in the diagnosis of HD patients who report shoulder pain.

## 1. Background

Chronic kidney disease (CKD) affects about 10–15% of the population worldwide [1]. End stage renal disease (ESRD) is the final permanent stage of CKD that represents a global public health issue and places a significant financial burden on healthcare systems [2]. In most countries, dialysis is the most prevalent form of kidney replacement therapy, with hemodialysis (HD) being the most commonly used modality [3]. Despite breakthroughs in HD therapy, long-term HD is associated with various complications including cardiovascular [4], neurological [5] and musculoskeletal system disorders [6].

Musculoskeletal pain is common among HD patients [7], and is typically caused by osteoarthritis or osteonecrosis as a result of abnormal bone and mineral metabolism, as well as extraskeletal calcifications [8, 9], and can be exacerbated by corticosteroid use during renal disease, with the knee, hip, and shoulder joints being the most commonly affected sites [7]. Additionally, the incidence of joint and soft tissue diseases may be caused by amyloid deposition that increases with the duration of HD therapy [10, 11].

Shoulder pain is frequently encountered in long-term hemodialysis patients [12]. The “dialysis shoulder” is a severe shoulder pain that occurs only when at rest, such as during HD or while sleeping, and is temporarily relieved by assuming the sitting position or rotating the shoulder joint. Although problems such as frozen shoulder and impingement syndrome might occur, discomfort at rest is the most common symptom [11]. Dialysis-induced b2-microglobulin amyloidosis has been linked to painful shoulders in HD patients [13]. The shoulder is one of the most often afflicted joints, affecting almost half of all patients on HD for more than 10 years [10, 14]. However, not all causes of shoulder pain are detectable on radiography; for example, tendon pathology cannot be seen on X-ray [15]. Although magnetic resonance imaging (MRI) is currently the gold standard for identifying shoulder disorders, it is time-consuming, expensive, and not always available. Also, patients with claustrophobia and MRI contraindications, such as pacemakers or cochlear implants, are unable to complete MRI examinations [16].

Musculoskeletal ultrasound (MSUS) is a well-established and useful method in the evaluation of shoulder pathologies, including rotator (tendon tears, tendinosis, and bursitis) and no rotator cuff abnormalities (e.g., synovial joint disorders and nerve entrapment syndromes) [17–21]. However, only a few research used MSUS to investigate shoulder pain in individuals with chronic renal failure on dialysis. The purpose of this study was to evaluate the validity of MSUS to properly detect shoulder disorders in HD patients with shoulder pain by identifying US anomalies in the shoulder and comparing them to those identified on MRI, with MRI serving as the gold standard test.

## 2. Methods

### 2.1. Patients

This cross-sectional observational study was conducted from January to June 2021 among 28 HD patients recruited from Mansoura Nephrology and Dialysis Unit (MNDU), Mansoura University Hospital, Egypt. The sample size was selected as a convenience sample; all patients who met the inclusion criteria were invited to participate in the study, unless they were excluded by any of the exclusion criteria or declined to participate. The criteria for selecting the subjects were as follows: (a) patients with ESRD on HD for more than 6 months, (b) age > 18 years, (c) shoulder pain either unilateral or bilateral for more than 6 weeks. The exclusion criteria were diabetes mellitus, autoimmune or connective tissue disease, pregnancy, history of surgery or trauma to the shoulder or chronic use of corticosteroids. Those with contraindications to MRI such as pacemakers, cochlear implants, or claustrophobia were excluded from the start.

Prior to being enrolled in the study, all participants signed an informed consent form. The ethical permission for this study was approved by the Mansoura Faculty of Medicine Institutional Research Board (approval number: MS.21.10.1690).

Demographic data, including age, gender, and residence was recruited. Other clinical characteristics such as duration since starting HD, laterality and duration of shoulder pain were also collected from each patient by interview.

### 2.2. Clinical Assessment of Both Shoulders

The biceps tendon (BT), acromioclavicular joint (ACJ), and subacromial-subdeltoid (SASD) bursa were all examined and palpated as part of a standard rheumatological examination of both shoulders. Flexion, extension, abduction, and internal and external rotation, as well as particular tests such as the Jobe's test [22] for supraspinatus tendon pathology and Yergason's test [23] for the long head of the BT, were all evaluated.

### 2.3. Shoulder Musculoskeletal Ultrasonography

Real-time ultrasound (US) scanning of both shoulders was performed using the EDAN U2 ultrasound device (Shenzhen, China) with a linear array transducer (8 to 13.4 MHz). The frequency was set to 13 MHz, and the sonographic parameters were tweaked to get the best US images of the shoulder structures. All sonographic examinations were carried out by a rheumatologist with at least 7 years of expertise in the field of MSUS. At the time of the evaluation, the operator was blinded to the clinical assessment of the patients. Shoulder scanning was done according to a set protocol that followed the technical guidelines of the European Society of Musculoskeletal Radiology [24]. The shoulder US protocol included examination of the rotator cuff and tendon of the long head of the biceps brachii muscle along the long and short axes, as well as the SASD bursa and ACJ. With the arm in external rotation, a posterior GHJ recess effusion was examined. Shoulder US static images and cine clips were retrospectively assessed by a rheumatologist experienced in acquiring and assessing MSUS images; with more than seven years of expertise in musculoskeletal imaging.

The MSUS images were collected for each patient and analysed later on with proprietary software. The US findings were interpreted using the Outcome Measures in Rheumatoid Arthritis Clinical Trials (OMERACT) US group definitions [25]. In the US, pathologic results were classified according to the following criteria: tendinosis/tendinopathy was characterized as tendon thickening accompanied by aberrant echogenicity and absence of the tendon's usual fibrillar echotexture. Distention of the biceps brachii tendon sheath was defined as hypoechoic or anechoic fluid or hypoechoic soft tissue surrounding the biceps tendon. SASD bursal thickening was described as a localized or diffuse bursal thickening of greater than 2 mm transverse thickness with or without bursal fluid. The presence of osteophytes with accompanying articular surface irregularity, with or without joint effusion or capsular thickening, was categorized as ACJ osteoarthritis.

To maximize specificity and remove false-positive diagnoses, doubtful or mild cases of tendinosis, osteoarthritis, and potential bursal thickening were excluded from the analysis.

### 2.4. Shoulder MRI

An MRI study was performed on a 1.5-T system (Ingenia, Phillips Healthcare) with an 8-channel shoulder array coil. Patient positioning was as follows: both shoulders of each patient were examined with the patient lying supine with both arms adducted in mild external rotation. All scans included protocol consisting of the following sequences; (1) coronal T1 FSE sequence (TR: 489 ms, TE: 20 ms, Matrix: 267 × 144, FOV: 200 mm, slice thickness: 3 mm), (2) coronal T2 FSE sequence (TR: 2885 ms, TE: 100 ms, Matrix: 267 × 144, FOV: 200 mm, slice thickness: 3 mm), (3) coronal STIR sequence (TR: 4978 ms, TE: 30 ms, Matrix: 267 × 144, FOV: 200 mm, slice thickness: 3 mm), (4) sagittal T2 FSE sequence (TR: 2885 ms, TE: 100 ms, Matrix: 267 × 144, FOV: 200 mm, slice thickness: 3 mm), (5) axial T2 FSE sequence (TR: 2885 ms, TE: 100 ms, Matrix: 267 × 144, FOV: 200 mm, slice thickness: 4 mm), and (6) axial GRE sequence (TR: 419 ms, TE: 14 ms, Flip angle: 25, Matrix: 267 × 144, FOV: 200 mm, slice thickness: 3 mm). The MR images were interpreted by two experienced radiologists in musculoskeletal MR imaging with more than eight-year experience. MRIs were assessed for any tendon abnormalities, joint effusion, humeral erosions, and ACJ osteoarthritis. The MRI results were analysed according to the following: tendinosis/tendinopathy was described as tendon thickening showing intrasubstance intermediate signal on T1 and T2 weighted images. Tenosynovitis was described as distention of the tendon sheath by fluid signal. SASD bursitis was described as distension of bursa by fluid signal. ACJ osteoarthritis was described as altered bone marrow signal at opposing articular margins, presence of osteophytes with accompanying articular surface irregularity, with or without joint effusion or capsular thickening.

### 2.5. Statistical Analysis

The collected data were coded, processed, and analysed using IBM SPSS for Windows version 24 (IBM Corp., Armonk, NY). Frequencies and relative percentages were used to present qualitative data. The mean and standard deviation were employed to represent quantitative data that was normally distributed, while the median, minimum, and maximum were used to represent quantitative data that was abnormally distributed. Comparisons between qualitative variables were conducted using the chi-square or Fisher exact tests, if necessary. The level of agreement between MSUS and both clinical examination and MRI of the shoulder was determined using the Kappa (*k*) coefficient. The agreement in this study was based on the kappa value, which is defined as *k* < 0.00 is “poor,” 0  <  *k* < 0.2 is “slight,” 0.21  <  *k* < 0.40 is “fair,” 0.41  <  *k* < 0.60 is “moderate,” 0.61  <  *k* < 0.80 is “substantial,” 0.81  <  *k* < 1.00 is “almost perfect”, and *k* = 1 is “perfect” agreement [26].

## 3. Results

Twenty-eight HD patients (16 males and 12 females) were recruited in the study with a mean age of 46.88 years. The median duration since starting HD was 3 years. Regarding shoulder pain, 18 patients (64.3%) had pain at one side while 10 patients (35.7%) had pain at both sides, with a median duration of 3 months, as shown in Table 1.

Rheumatological examination of 38 symptomatic shoulders was suggestive of the following pathologies: BT tenosynovitis in 10 (26.3%), subscapularis tendinopathy in 26 (68.4%), supraspinatus tendinopathy in 14 (36.8%), infraspinatus tendinopathy in 10 (26.3%), shoulder impingement syndrome in 10 (26.3%), and ACJ pathology in 12 (31.6%). Findings from MSUS and MRI of symptomatic and asymptomatic shoulders were illustrated in Table 2.

US abnormalities were prevalent in almost all patients. Supraspinatus tendinopathy was the most common abnormality in symptomatic shoulders (92.1%), followed by SASD bursitis (65.8%), humoral erosions (57.9%), and ACJ osteoarthritis (52.6%). On the other hand, supraspinatus tendinopathy and SASD bursitis were detected in more than one half of the examined asymptomatic shoulders (66.7% and 55.6%, respectively) (Table 2).

Tables 3 and 4 show the performance of clinical evaluation when compared to MSUS findings of the shoulder. There was poor agreement between them in detecting almost all shoulder pathologies.

On MRI examination, supraspinatus tendinopathy was the most common abnormality in both asymptomatic and symptomatic shoulders (77.8% vs 94.7%, respectively, *p*=0.05). There was a statistically significant difference between symptomatic and asymptomatic shoulders regarding the presence of BT tenosynovitis (*p*=0.01) and supraspinatus tendinopathy (*p*=0.05).

**Figure 1:**
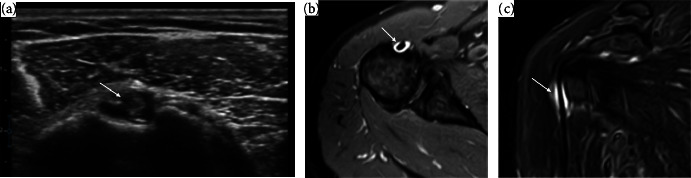
US and MRI findings of 50-year-old female patient with 4-year hemodialysis duration, presenting with shoulder pain for 6 month. (a) US scan transverse view of biceps tendon shows a hypoechoic area around the biceps tendon (arrow) denoting tenosynovitis. (b & c) MRI shoulder axial &coronal STIR images revealed mild amount fluid SI within synovial sheath of biceps tendon (arrow) denoting biceps tenosynovitis.

**Figure 2:**
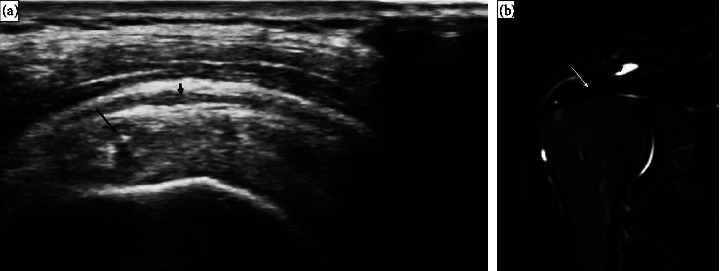
Ultrasound and MRI findings in a 46-year-old male patient with a 5-year history of hemodialysis with unilateral shoulder pain for 6 month. (a) US scan of supraspinatus tendon on its long axis shows thickness of the tendon with loss of its normal fibrillar pattern (long arrow) with minimal fluid collection at the subacromion subdeltoid bursa (short arrow) denoting supraspinatus tendinosis and subacromion subdeltoid bursitis. (b) MRI shoulder coronal STIR images revealed subacromion subdeltoid bursitis (short arrow) and supraspinatus tendinosis (long arrow).

The diagnostic accuracy of MSUS, in relation to the gold standard MRI examination, in detecting shoulder abnormalities was presented in Table 5 and illustrated in Figures 1 and 2. MSUS shows high sensitivity and specificity in all the studied shoulder pathologies except GHJ effusion (sensitivity, 33.3%) and infraspinatus tendinopathy (sensitivity, 58.3%). The percentage of agreement between MSUS and MRI in detecting BT tenosynovitis was 82.14% (kappa, 0.64), subscapularis tendinopathy 83.93% (kappa, 0.654), supraspinatus tendinopathy 91.07% (kappa, 0.617), infraspinatus tendinopathy 82.14% (0.470), SASD bursitis 80.36% (kappa, 0.569), humeral head erosions 82.14% (kappa, 0.635), GHJ effusion 82.14% (kappa, 0.352), and ACJ osteoarthritis 76.79% (kappa, 0.539), as shown in Table 6.

## 4. Discussion

Patients on HD may experience a limited range of shoulder movement as well as a significant loss of muscle strength [27]. Abduction of the arm, which has a relatively limited range of motion, causes bilateral shoulder pain. The discomfort is intensified when lying in a supine posture, such as during sleep, making the dialysis session difficult to tolerate. Physical examination may reveal the “shoulder pad sign,” in which the shoulders affected by dialysis-related amyloidosis appear hypertrophied due to thickness and/or amyloid deposition between the rotator cuff muscles and tendons. MSUS, which can detect pads deposited between muscles and tendons, can be used to confirm the thickness of the rotator cuff [28]. The supraspinatus and/or subscapularis tendon could easily be seen thickened on magnetic resonance imaging. It is possible that arm tendons are implicated in b2-microglobulin deposition; the bicipital tendon becomes painful to touch, which can be seen on US or MRI.

To the best of our knowledge, this is the first study to investigate whether MSUS, a simple bedside, noncostly and time-saving technique, can substitute MRI in assessing shoulder pathology in HD patients. In the current study, US abnormalities were prevalent in almost all patients. Supraspinatus tendinopathy was the most frequently detected abnormality in symptomatic shoulders (92.1%), followed by SASD bursitis (65.8%), humoral erosions (57.9%), and ACJ osteoarthritis (52.6%). In the same line, supraspinatus thickening was identified at significantly higher rates in symptomatic HD patients in an MSUS study by Barisic et al., which included 54 hemodialysis patients and 50 healthy controls [29]. Sommer and colleagues used MSUS to examine 14 shoulders of patients who had been on HD for at least 10 years and found biceps tendinitis, supraspinatus tendinitis, SASD bursitis, and supraspinatus rupture in 50%, 27.5%, 35.7%, and 7%, respectively [28].

Supraspinatus tendinopathy was the most prevalent abnormality in MRI in both asymptomatic and symptomatic shoulders in the current study (77.8% vs 94.7%, respectively, *p*=0.5). Similarly, Turk et al. used MRI shoulder to compare HD patients with and without shoulder pain and found that supraspinatus thickness was higher in the former group [30].

In Escobedo et al. work, significant thickening in the supraspinatus was detected when all symptomatic patients (*n*: 5, more than 10 years HD) were compared to asymptomatic patients who received dialysis for a shorter period (*n*: 4, 5 years HD). A 20% increase in thickness was seen in asymptomatic individuals as compared to normal/healthy adults, indicating early amyloid accumulation. In pathological samples from tendon and capsular materials of individuals with dialysis-related amyloidosis, thickening in the supraspinatus tendon was proven to be a considerably significant indicator of amyloid deposition in MRI [31].

MRI observations of dialysis-related shoulder arthropathy have included thickening of the supraspinatus and subscapular tendons, aberrant liquid collection in the joint and bursa, and periarticular osseous lesions [32–34].

In the present study, there was poor agreement between clinical evaluation and MSUS in detecting almost always all shoulder pathologies. The agreement between clinical assessment and MSUS at the shoulders was relatively poor. As a result, MSUS can provide clinical examinations with additional data [35]. MSUS has been shown to be more sensitive and accurate than clinical examination in detecting and scoring erosions and inflammatory and degenerative changes than clinical examination [36, 37].

On the other hand, MSUS had a high sensitivity and specificity in detection of all of the shoulder pathologies evaluated in our study, with the exception of GHJ effusion (sensitivity, 33.3%) and infraspinatus tendinopathy (sensitivity, 58.3%). The percentage of agreement between MSUS and MRI in detecting biceps tenosynovitis was 82.14% (kappa, 0.64), subscapularis tendinopathy 83.93% (kappa, 0.654), supraspinatus tendinopathy 91.07% (kappa, 0.617), infraspinatus tendinopathy 82.14% (0.470), SASD bursitis 80.36% (kappa, 0.569), humeral head erosions 82.14% (kappa, 0.635), GHJ effusion 82.14% (kappa, 0.352), and ACJ osteoarthritis 76.79% (kappa, 0.539). In a similar study, the accuracy of US detection of rotator cuff and BT integrity was comparable to that of MRI [16].

In another prospective study of 50 patients who were referred for MSUS and MRI because of shoulder discomfort, 6 (12%) of the patients had BT sheath effusion on MSUS, while 7 (14%) had BT sheath effusion on MRI. As a consequence, there was a high degree of agreement between MSUS and MRI in diagnosing BT sheath effusion [38]. In addition, Alasaarela et al. found effusion of the BT sheath in 24 and 20 shoulders on MRI and USG, respectively, indicating the significant agreement between the two modalities in identifying such a lesion [39]. Similar findings were reported by McMonagle and Vinson [40] and Fischer et al. [16], who concluded that MSUS and MRI were equivalent and that MSUS was useful in revision scenarios. In another study [16], the pathology in the supraspinatus was detected with 91.1% accuracy, the infraspinatus with 84.4% accuracy, and the SSC tendon with 77.8% accuracy.

One of the study's strengths is that it provides a thorough examination of symptomatic and asymptomatic shoulders in HD patients. Our research, however, has certain limitations. The most important limitation is the relatively small number of patients. The lack of a control group, as only HD patients were included, is a second important limitation. The fact that we only categorized MSUS results as present or absent ads to the study's limitations. To graduate and score, these MSUS findings might be helpful to obtain more detailed descriptions. Additionally, one single examiner performed the MSUS examination. The evaluation of MSUS scans by another expert examiner would ensure the consistency of this study. Lastly, because a diagnosis is frequently an overall judgement made by the examiner based on the findings of pathological abnormalities in the images, the research should compare the image findings and not the examiner's or MRI radiologist's diagnosis. Based on our data, we may conclude that shoulder problems are common in HD patients, even in people who do not have obvious shoulder complaints. MSUS is a valuable imaging technique that assists in the diagnosis and management of many HD patients who report shoulder discomfort. It is also a good modality for assessing shoulder pathology in HD patients because of its wide availability, low cost, and tolerability. MRI may be beneficial for patients whose MSUS results are ambiguous.

## Figures and Tables

**Table 1:** Demographic and clinical data in the study HD patients (*n* = 28).

Variable	HD patients (*n* = 28)
Age (years)	46.89 ± 15.99
*Age groups (years)*	
20–39	10 (35.7)
≥40	18 (64.3)

*Gender*	
Male	16 (57.1)
Female	12 (42.9)

*Residence*	
Rural	18 (64.3)
Urban	10 (35.7)
Years since starting dialysis	3 (0.5–14)

*Shoulder pain*	
Unilateral	18 (64.3)
Bilateral	10 (35.7)

Duration of shoulder pain, months	6 (1–24)

Data were expressed in mean ± SD, *n* (%) or median (min-max).

**Table 2:** Ultrasonographic and MRI findings in asymptomatic (*n* = 18) and symptomatic shoulders (*n* = 38) in the study HD patients.

Variable	Ultrasonographic findings			MRI findings		
	Asymptomatic shoulders (*n* = 18) *n* (%)	Symptomatic shoulders (*n* = 38) *n* (%)	*P*	Asymptomatic shoulders (*n* = 18) *n* (%)	Symptomatic shoulders (*n* = 38) *n* (%)	*P*
Biceps tenosynovitis	2 (11.1)	19 (50)	**0.007** ^*∗*^	4 (22.2)	23 (60.5)	**0.010** ^*∗*^
Subscapularis tendinosis	5 (27.8)	16 (42.1)	0.301	8 (44.4)	12 (31.6)	0.348
Supraspinatus tendinopathy	12 (66.7)	35 (92.1)	**0.015** ^*∗*^	14 (77.8)	36 (94.7)	**0.050** ^*∗*^
Infraspinatus tendinopathy	2 (11.1)	10 (26.3)	0.300	6 (33.3)	6 (15.8)	0.135
SASD bursitis	10 (55.6)	25 (65.8)	0.460	13 (72.2)	25 (65.8)	0.630
Humeral erosions	4 (22.2)	22 (57.9)	**0.021** ^*∗*^	4 (22.2)	16 (42.1)	0.233
Glenohumeral effusion	0	6 (15.8)	—	4 (22.2)	8 (21.1)	1
Acromioclavicular osteoarthritis	6 (33.3)	20 (52.6)	0.176	8 (44.4)	23 (60.5)	0.388

SASD: subacromial subdeltoid ^*∗*^*P* < 0.05.

**Table 3:** Comparison of physical examination versus ultrasound in detecting shoulder pathologies in HD patients (*n* = 56 shoulders).

Variable	Sensitivity (%)	Specificity (%)	PPV (%)	NPV (%)	Accuracy (%)
Biceps tenosynovitis	50	65.2	23.8	85.7	62.5
Subscapularis tendinosis	53.6	78.6	71.4	62.9	66.1
Supraspinatus tendinopathy	100	21.4	19.1	100	41.1
Infraspinatus tendinopathy	21.4	80.4	25	84.1	71.4

**Table 4:** Agreement between clinical examination and ultrasound in detecting shoulder pathologies in the study HD patients (*n* = 56 shoulders).

Variable	Kappa agreement	SE	*P*	Percentage of observed agreement (%)	Percentage of agreement by chance (%)	Strength of agreement
Biceps tenosynovitis	0.106	0.123	0.368	62.5	58	Poor
Subscapularis tendinosis	0.321	0.123	**0.013** ^*∗*^	66	50	Fair
Supraspinatus tendinopathy	0.120	0.047	0.059	41.1	33	Poor
Infraspinatus tendinopathy	0.097	0.145	0.466	71.4	68.3	Poor
Acromioclavicular osteoarthritis	0.181	0.113	0.114	60.7	52	Poor

^*∗*^ *P* < 0.05.

**Table 5:** Comparison of ultrasound versus magnetic resonance in detecting shoulder pathologies in the study HD patients (*n* = 56 shoulders).

Variable	Sensitivity (%)	Specificity (%)	PPV (%)	NPV (%)	Accuracy (%)
Biceps tenosynovitis	70.4	93.1	90.5	77.1	82.1
Subscapularis tendinosis	80	86.1	76.2	88.6	83.9
Supraspinatus tendinopathy	92	83.3	97.9	55.6	91.1
Infraspinatus tendinopathy	58.3	88.6	58.3	88.6	82.1
SASD bursitis	81.6	77.8	88.6	66.7	80.4
Humeral erosions	90	77.8	69.2	93.3	82.1
Glenohumeral effusion	33.3	95.5	66.7	84	82.1
Acromioclavicular osteoarthritis	71	84	84.6	70	76.8

**Table 6:** Agreement between ultrasound and magnetic resonance imaging in detecting shoulder pathologies in the study HD patients (*n* = 56 shoulders).

Variable	Kappa agreement	SE	*P*	Percentage of observed agreement (%)	Percentage of agreement by chance (%)	Strength of agreement
Biceps tenosynovitis	0.64	0.101	**<0.001** ^*∗*^	82.14	61.29	Substantial
Subscapularis tendinosis	0.654	0.105	**<0.001** ^*∗*^	83.93	53.57	Substantial
Supraspinatus tendinopathy	0.617	0.154	**<0.001** ^*∗*^	91.07	76.66	Substantial
Infraspinatus tendinopathy	0.470	0.143	**<0.001** ^*∗*^	82.14	66.33	Moderate
SASD bursitis	0.569	0.115	**<0.001** ^*∗*^	80.36	54.46	Moderate
Humeral erosions	0.635	0.102	**<0.001** ^*∗*^	82.14	51.02	Substantial
Glenohumeral effusion	0.352	0.156	**0.004** ^*∗*^	82.14	72.45	Fair
Acromioclavicular osteoarthritis	0.539	0.11	**<0.001** ^*∗*^	76.79	49.62	Moderate

^*∗*^ *P* < 0.05.

## Data Availability

The datasets used and/or analysed during the current study are available from the corresponding author upon reasonable request.

## Abbreviations

ACJ: Acromioclavicular joint
BT: Biceps tendon
CKD: Chronic kidney disease
ESRD: End stage renal disease
GHJ: Glenohumeral joint
HD: Hemodialysis
MRI: Magnetic resonance imaging
MSUS: Musculoskeletal ultrasound
OMERACT: Outcome Measures in Rheumatoid Arthritis Clinical Trials
SASD: Subacromial-subdeltoid.
